# Supplementary material for: Effects of therapeutic hypothermia on death among asphyxiated neonates with hypoxic-ischemic encephalopathy: A systematic review and meta-analysis of randomized control trials
Source: PLoS One. 2021 Feb 25;16(2):e0247229. doi: 10.1371/journal.pone.0247229 (PMC7906350; doi:10.1371/journal.pone.0247229)
Supplement: S3 Fig — (DOCX) [file pone.0247229.s004.docx]

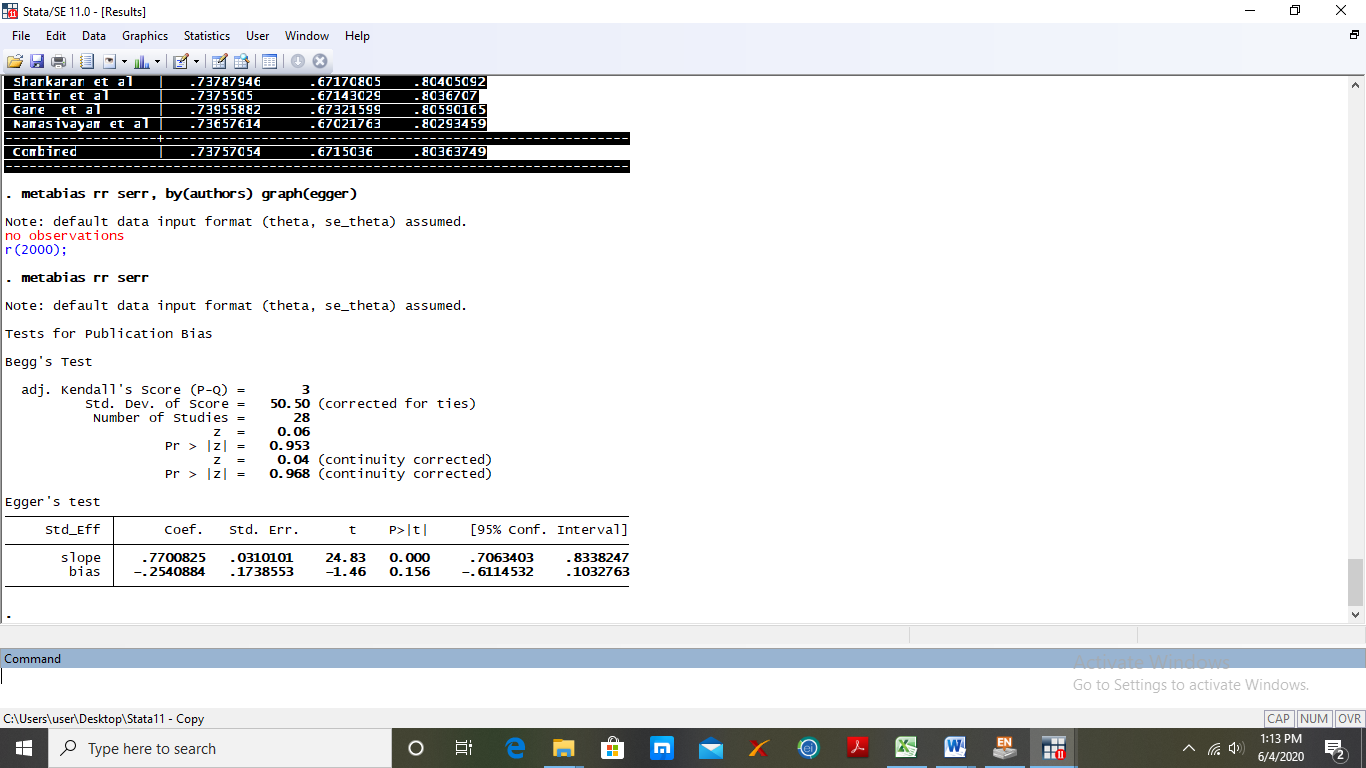


S3 Fig: Eggers test showing publication bias on the effects of therapeutic hypothermia on death among asphyxiated neonates with hypoxic ischemic encephalopathy
