# Supplementary material for: Effects of therapeutic hypothermia on death among asphyxiated neonates with hypoxic-ischemic encephalopathy: A systematic review and meta-analysis of randomized control trials
Source: PLoS One. 2021 Feb 25;16(2):e0247229. doi: 10.1371/journal.pone.0247229 (PMC7906350; doi:10.1371/journal.pone.0247229)
Supplement: S1 Table — (DOCX) [file pone.0247229.s005.docx]

# S1 Table. Search strategy used for one of the databases

| Medline/PubMed | | |  |
| --- | --- | --- | --- |
|  | **Search terms** | |  |
| Group | **Non-MeSH terms** | **MeSH (sub-terms in MeSH)** | **Citations** |
| #1 | new-borns  perinatal  infant | Neonates |  |
| #2 | Cooling  Temperature  body temperature | hypothermia |  |
| #3 | Mortality | death |  |
|  |  |  |  |
| #4 | asphyxia  hypoxia  brain  encephalopathy | hypoxic-ischemic |  |
| #5 | Therapy |  |  |
| #1 AND #2 AND #3 AND #4 AND #5 |  |  | **2252** |

(Neonate [MeSH Terms] OR newborn OR perinatal OR infant) AND (hypothermia [MeSH Terms] OR cool OR cooling OR temperature OR body temperature) AND (death [MeSH Terms] OR mortality) AND (asphyxia [MeSH Terms] OR hypoxic-ischemic OR hypoxic-ischaemic OR hypoxia OR brain OR encephalopathy AND therapy
