# Supplementary material for: Effects of therapeutic hypothermia on death among asphyxiated neonates with hypoxic-ischemic encephalopathy: A systematic review and meta-analysis of randomized control trials
Source: PLoS One. 2021 Feb 25;16(2):e0247229. doi: 10.1371/journal.pone.0247229 (PMC7906350; doi:10.1371/journal.pone.0247229)
Supplement: S2 Table — (DOCX) [file pone.0247229.s006.docx]

S2 Table: Quality appraisal result of included studies; Using Joanna Briggs Institute (JBI) quality appraisal checklist

| **Author** | **Quality assessment questions** | | | | | | | | | | |  |  |  |  |  |
| --- | --- | --- | --- | --- | --- | --- | --- | --- | --- | --- | --- | --- | --- | --- | --- | --- |
|  | Q1 | Q2 | Q3 | Q4 | Q5 | Q6 | Q7 | Q8 | Q9 | Q10 | Q11 | Q12 | Q13 | Yes Total | Quality status | Overall appraisal |
| Akisu M et al | Y | Y | Y | Y | Y | Y | Y | Y | Y | Y | Y | Y | Y | 13/13 | Low risk | Included |
| Lin ZL et al | Y | Y | Y | Y | Y | Y | UN | Y | Y | Y | Y | Y | Y | 12/13 | Low risk | Included |
| Zhou WH et al | Y | Y | Y | Y | Y | Y | Y | Y | Y | Y | Y | Y | Y | 13/13 | Low risk | Included |
| Robertson et al | Y | Y | Y | Y | Y | UC | UC | Y | Y | Y | Y | Y | Y | 11/13 | Low risk | Included |
| Thayyil S et al | Y | Y | Y | Y | Y | Y | UC | Y | Y | Y | Y | Y | Y | 12/13 | Low risk | Included |
| Bharadwaj et al | Y | Y | UC | Y | Y | N | UC | Y | Y | Y | Y | Y | Y | 11/13 | Low risk | Included |
| Bhat M et al | Y | Y | Y | Y | Y | Y | Y | Y | Y | Y | N | Y | Y | 13/13 | Low risk | Included |
| Azzoparadi et al | Y | Y | Y | Y | Y | Y | UC | Y | Y | Y | Y | Y | Y | 12/13 | Low risk | Included |
| Jacobs et al | Y | Y | Y | Y | Y | Y | Y | Y | Y | Y | Y | Y | Y | 12/13 | Low risk | Included |
| Shankaran et al | Y | Y | Y | Y | Y | Y | UC | Y | Y | Y | Y | Y | Y | 12/13 | Low risk | Included |
| Simbruner et al | Y | Y | Y | Y | Y | UC | UC | Y | Y | Y | Y | Y | Y | 11/13 | Low risk | Included |
| Gluckman et al | Y | Y | Y | Y | Y | Y | Y | Y | Y | Y | Y | Y | Y | 13/13 | Low risk | Included |
| Zhou et al | Y | Y | Y | Y | Y | Y | Y | Y | Y | Y | Y | Y | Y | 12/13 | Low risk | Included |
| Eicher DJ et al | Y | Y | Y | Y | Y | Y | Y | Y | Y | Y | N | Y | Y | 13/13 | Low risk | Included |
| Battin MR et al | Y | Y | Y | Y | Y | Y | Y | Y | Y | UC | N | Y | Y | 12/13 | Low risk | Included |
| Shankaran et al | Y | Y | Y | Y | Y | Y | Y | Y | Y | Y | Y | Y | Y | 13/13 | Low risk | Included |
| Joy R et al | Y | Y | Y | Y | Y | Y | UC | Y | UC | Y | Y | Y | Y | 11/13 | Low risk | Included |
| Maoulainine et al | Y | Y | Y | Y | Y | Y | Y | Y | Y | Y | Y | Y | Y | 13/13 | Low risk | Included |
| Laptook AR et al | Y | Y | Y | Y | Y | N | UC | Y | Y | Y | Y | Y | Y | 12/13 | Low risk | Included |
| Gane B. D et al | Y | Y | Y | Y | Y | Y | Y | Y | Y | Y | Y | Y | Y | 13/13 | Low risk | Included |
| Selway L et al | Y | Y | Y | Y | Y | Y | UC | Y | Y | Y | Y | Y | Y | 12/13 | Low risk | Included |
| Susan E. et al | Y | Y | Y | Y | Y | Y | Y | Y | Y | Y | Y | Y | Y | 13/13 | Low risk | Included |
| Jose S et al | Y | Y | Y | Y | Y | Y | Y | Y | UC | Y | Y | Y | Y | 12/13 | Low risk | Included |
| Azzopardi MD et al | Y | Y | Y | Y | Y | UC | UC | Y | Y | Y | UC | Y | Y | 10/13 | Low risk | Included |
| Shankaran, MD et al | Y | Y | Y | Y | Y | UC | Y | Y | UC | Y | UC | Y | Y | 10/13 | Low risk | Included |
| Battin, M. R et al | N | Y | Y | Y | Y | UC | Y | Y | Y | Y | UC | Y | Y | 11/13 | Low risk | Included |
| Gane, B. D et al | Y | Y | UC | Y | Y | Y | UC | Y | UC | Y | Y | Y | Y | 10/13 | Low risk | Included |
| Namasivayam A et al | Y | Y | Y | Y | Y | UC | Y | Y | Y | Y | UC | Y | Y | 11/13 | Low risk | Included |

Key: Y=yes, N=no, UC=unclear, Q=Question

***JBI Critical Appraisal Checklist for Randomized Controlled Trials***

*Q1. Was true randomization used for assignment of participants to treatment groups?*

*Q2. Was allocation to treatment groups concealed?*

*Q3. Were treatment groups similar at the baseline?*

*Q4. Were participants blind to treatment assignment?*

*Q5. Were those delivering treatment blind to treatment assignment?*

*Q6. Were outcomes assessors blind to treatment assignment?*

*Q7. Were treatment groups treated identically other than the intervention of interest?*

*Q8. Was follow up complete and if not, were differences between groups in terms of their follow up adequately described and analyzed?*

*Q9. Were participants analyzed in the groups to which they were randomized?*

*Q10. Were outcomes measured in the same way for treatment groups?*

*Q11. Were outcomes measured in a reliable way?*

*Q12. Was appropriate statistical analysis used?*

*Q13. Was the trial design appropriate, and any deviations from the standard RCT design (individual randomization, parallel groups) accounted for in the conduct and analysis of the trial?*
