# Supplementary material for: Effects of therapeutic hypothermia on death among asphyxiated neonates with hypoxic-ischemic encephalopathy: A systematic review and meta-analysis of randomized control trials
Source: PLoS One. 2021 Feb 25;16(2):e0247229. doi: 10.1371/journal.pone.0247229 (PMC7906350; doi:10.1371/journal.pone.0247229)
Supplement: S1 Synopsis — (DOCX) [file pone.0247229.s007.docx]

**Synopsis**

**Research question:** Does therapeutic hypothermia reduce death among asphyxiated neonates with hypoxic-ischemic encephalopathy?

**What’s already known:** Previous randomized control trials conducted across the world reported the relative risk of mortality among birth asphyxiated neonates with hypoxic-ischemic encephalopathy after the implementation of therapeutic hypothermia. The relative risk of mortality in such randomized control trials ranged from 0.00 ([16](#_ENREF_16)) to 0.95 ([17](#_ENREF_17)). There is an inconsistency report on the relative risk of mortality across different countries in the world

**What this study adds:** Therapeutic hypothermia reduces the risk of death in neonates with moderate to severe hypoxic-ischemic encephalopathy. Both selective head cooling and whole-body cooling method are effective in reducing the mortality of infants with this condition. Moreover, low income countries benefit the most from the therapy.
