# Supplementary material for: Effects of therapeutic hypothermia on death among asphyxiated neonates with hypoxic-ischemic encephalopathy: A systematic review and meta-analysis of randomized control trials
Source: PLoS One. 2021 Feb 25;16(2):e0247229. doi: 10.1371/journal.pone.0247229 (PMC7906350; doi:10.1371/journal.pone.0247229)
Supplement: Attachment — Submitted filename: Response to Reviewers.docx [file pone.0247229.s008.docx]

| Date: | 9/24/2020 |
| --- | --- |
| To: | "PLOS ONE" plosone@plos.org |
| From: | "Biruk Beletew Abate" birukkelemb@plos.com |
| Subject: | Point to point response / 'Response to Reviewers'. |

PONE-D-20-24388
Effects of therapeutic hypothermia on death among asphyxiated neonates with hypoxic-ischemic encephalopathy: a systematic review and meta-analysis of randomized control trials
PLOS ONE
**To Editor**
Dear Academic Editor (Georg M. Schmölzer) we have no word to explain our deepest thanks for your constructive comments and helping us throughout the process. Since we have agreed with all points you raised we believe we have carefully amended the paper as per your point of view. We have presented the point to point response below. Thank you again since you have contributed much for our better paper.

**Editor comment 1.** Please ensure that your manuscript meets PLOS ONE's style requirements, including those for file naming. The PLOS ONE style templates can be found at

https://journals.plos.org/plosone/s/file?id=wjVg/PLOSOne_formatting_sample_main_body.pdf and https://journals.plos.org/plosone/s/file?id=ba62/PLOSOne_formatting_sample_title_authors_affiliations.pdf

**Authors’ response:** Thanks for supplying us the PLOS ONE style templates and we make sure the manuscript is as per these templates.

**Editor comment** 2. Please include your tables as part of your main manuscript and remove the individual files. Please note that supplementary tables (should remain/ be uploaded) as separate "supporting information" files.

**Authors’ response:** We have included tables and figures as part of the manuscript and we have uploaded the supplementary tables and figures as separate "supporting information" files as per your recommendation.

**Editor comment 3**. Please include a caption for figure 4.

**Authors’ response:** We have included as per your comment

**Editor comment** 4. Please include captions for your Supporting Information files at the end of your manuscript, and update any in-text citations to match accordingly. Please see our Supporting Information guidelines for more information: [http://journals.plos.org/plosone/s/supporting-information](about:blank).

**Authors’ response:** we have included captions for your Supporting Information files at the end of the manuscript

 **To Reviewer #1:**

Dear Ola Didrik Saugstad (Reviewer) we would like to forward our deep-seated gratitude for your interesting and valuable comments. We all really appreciate your potential and optimism while you give such constructive and in-depth comments. Since we have agreed with all of your points raised we believe we have amended the manuscript as per your comments. We would like to thank you again since you are contributing for our better paper by giving such comments which is important to improve the quality of this paper. Below we have written the point to point response to issues you raised.

**Reviewer comment:** This meta-analysis and systematic review by Biruk Beletew et al regarding effect of hypothermia on mortality in HIE newborn babies is important and timely. The authors basically confirm previous meta-analyses demonstrating a 25% reduction in mortality after cooling. However, the authors conclude that cooling is most efficient in low income countries. If this is correct, it is important because until recently there has been concerns regarding cooling in such areas due to higher mortality. Further, the authors find similar effects using head cooling and total body cooling.
I have some comments and concerns:
**Reviewer comment:** 1.Introduction first sentence: Perhaps it is more appropriate to write: intrapartum and neonatal asphyxia. A modern term of birth asphyxia is however adverse intrapartum events

**Authors’ response:** we amended the sentence as per your comment: Introduction; page 3 line 50 and 51.

**Reviewer comment:** 2. In the introduction the authors should mention that a powerful mean to reduce HIE is to avoid hyperoxia. See for instance:

Kapadia VS, Chalak LF, DuPont TL, Rollins NK, Brion LP, Wyckoff MH. Perinatal asphyxia with hyperoxemia within the first hour of life is associated with moderate to severe hypoxic-ischemic encephalopathy. J Pediatr. 2013;163(4):949-954.

Saugstad OD. The oxygen paradox in the newborn: keep oxygen at normal levels. J Pediatr. 2013;163(4):934-935.

and reducing death:
Saugstad OD, Ramji S, Soll RF, Vento M. Resuscitation of newborn infants with 21% or 100% oxygen: an updated systematic review and meta-analysis. Neonatology. 2008;94(3):176-182.

**Authors’ response:** We really thank you for providing us such very important papers related to our manuscript. We have downloaded all the above references, used and cited in the introduction part of the manuscript as per your recommendation.

**Reviewer comment:** 3. It would be useful to separate data in studies in which the newborns were resuscitated with 100% O2 (approx before 2010) and with air.
**Authors’ response:** we included those studies which have similar resuscitation status which is the standard treatment including 21 % O2. That is why we didn’t conduct subgroup analysis by level of resuscitation separately.
**Reviewer comment:** 4. I have problems with some of the results. The authors find in general a reduced relative risk of death (RR 0.74). That is a 26 % reduction. how can this be turned to a 75% reduced risk of death in the discussion part? See: "The pooled relative risk of mortality among birth asphyxiated neonates who have got cooling therapy was found to be nearly 75% lower compared with those who haven’t got cooling therapy." Please explain
**Authors’ response:** It was by mistake we said 75%. Now we have amended it to 26%. Sorry for our mistake and thanks since you remembered us.

**Reviewer comment:** 5. Regarding the results from low income countries two studies seem to be included. One of these is naturally Robertson NJ et al from Uganda 2008. In this study there was an approiximate 6 fold increased risk of death in the cooled babies compared to non cooled. (There was also more seizures in the cooled group). The Robertson study is small, still, based on this how is it possible to get a RR of 0.32 for death in low income countries ? The Robertson study has been very important for recommendations of being cautious using cooling in low income areas.
**Authors’ response:** Regarding the Robertson study; the authors taken small number of studies which was 21:15 hypothermic and standard treatment respectively. As a result during pooling we are doing a weighted mean , that means studies which have large sample size will have high contribution on the pooled effect while studies like Robertson will have small contribution. While the other study, Maullanie et al, in low income country have a larger sample size and the RR is 0.43. That is why RR of 0.32 for death in low income which is near to 0.43.

**Reviewer comment:** Conclusion

This study has many merits. It is well performed and well written. It deals with a highly important topic. My main objection is that I don’t understand all the results. I look forward to a clarification by the authors.

**Authors’ response:** we really thank you for the above constructive comments. Regarding the results; since, this systematic review and meta-analysis aimed to assess the effect of therapeutic hypothermia on death among asphyxiated neonates with hypoxic-ischemic encephalopathy.

As a result we extracted the author's name, year of publication, country, method of cooling, the severity of encephalopathy, the sample size in the hypothermic, and non-hypothermic groups, and **the number of deaths in the intervention and control groups**. The primary outcome was the **relative risk (RR) of death**, which was calculated as **the proportion of death among hypothermic over the proportion of death among non-hypothermic patients.** We calculated the RR for each included studies. Finally we pooled the estimates RR of death. The pooled relative risk of mortality after implementation of therapeutic hypothermia was found to be 0.74 (95%CI; 0.67, 0.80; I^2^=0.0%; p<0.996). That means a 26% reduction in mortality is revealed among cooled (hypothermic) groups compared to non-hypothermic groups. The relative risk of mortality was the same in selective head cooling and whole-body cooling method which was 0.74. Regarding the cooling device, the pooled relative risk of mortality is the same between the cooling cap and cooling blanket (0.74).
