# Supplementary material for: Effects of therapeutic hypothermia on death among asphyxiated neonates with hypoxic-ischemic encephalopathy: A systematic review and meta-analysis of randomized control trials
Source: PLoS One. 2021 Feb 25;16(2):e0247229. doi: 10.1371/journal.pone.0247229 (PMC7906350; doi:10.1371/journal.pone.0247229)
Supplement: Attachment — Submitted filename: Response to reviewer.docx [file pone.0247229.s009.docx]

PONE-D-20-24388R1
Effects of therapeutic hypothermia on death among asphyxiated neonates with hypoxic-ischemic encephalopathy: a systematic review and meta-analysis of randomized control trials
PLOS ONE

| Date: | 9/24/2020 |
| --- | --- |
| To: | "PLOS ONE" plosone@plos.org |
| From: | "Biruk Beletew Abate" birukkelemb@plos.com |
| Subject: | Point to point response / 'Response to Reviewers'. |

PONE-D-20-24388R1
***Effects of therapeutic hypothermia on death among asphyxiated neonates with hypoxic-ischemic encephalopathy: a systematic review and meta-analysis of randomized control trials***
PLOS ONE
**To Editor**
Dear Academic Editor (Georg M. Schmölzer) we have no word to explain our deepest thanks for your constructive comments and helping us throughout the process. We have added one Author who fulfill the authorship criteria of the journal whom the corresponding author forgot during submission of the manuscript. For this we ask apologize. Thank you again since you have contributed much for our better paper.

**To Reviewer #1:**

Dear Ola Didrik Saugstad (Reviewer) we would like to forward our deep-seated gratitude for your interesting and valuable comments. We all really appreciate your potential and optimism while you give such constructive and in-depth comments. Since we have agreed with all of your points raised we believe we have amended the manuscript as per your comments. We would like to thank you again since you contributed much the quality of this paper. Below we have written the point to point response to indicate we addressed the issues you raised.

**Reviewer comment:**: This manuscript has been much improved and I want to congratulate the authors. I still have some comments

**Authors’ response:** thanks since you recognized our effort, we have again amended the manuscript considering all your comments.

**Reviewer comment:** 1. I am still concerned regarding the results of low income countries. Only two studies are available and listed, Robertson et al and Maoulainine et al. Robertson found higher mortality with hypothermia while Maoulainine et al found the opposite. Could the authors explain to me that in the table Robertson et al has an ES (is this relative risk?) of 0.10 while Maoulainine et al show 0.43. The fact is that Robertson's results are far above 1 although not significant (5. 0, 95 %CI 0,7-37). Maoulainine et al's study from Morrocco includes 38 babies (19 in each group) while Robetson et al include 36 babies , of these 21 treated with cooling and 15 received standard care. The size of these two studies therefore are quite similar.
**Authors’ response:** as you explained from low income countries only two studies were included because these are the only studies which included the **outcome of interest** and fulfill the study’s **inclusion criteria**. However, you are correct this is the **limitation of this study**. Regarding the estimate in the two studies, you are again correct the Robertson's results not significant (5. 0, 95 % CI 0,7-37).

For purpose of clarity, in Robertson's results the proportion of death among neonates with therapeutic hypothermia and standard treatment were 1/21and 7/15 respectively. Then the relative risk of death among neonates with cooling therapeutic becomes 0.10(*Robertson NJ, Nakakeeto M, Hagmann C, Cowan FM, Acolet D, Iwata O, et al. Therapeutic hypothermia for birth asphyxia in low-resource settings: a pilot randomised controlled trial. The Lancet. 2008;372(9641):801-3*). Meanwhile, Maoulainine et al's study from Morrocco includes 38 babies (19 in each group) 3:7 the proportion of death among neonates with theraputhic hypothermia and standard treatment were 3/19 and 7/19 respectively. Then the relative risk of death among neonates with cooling therapeutic becomes 0.43 *(Maoulainine F, Elbaz M, Elfaiq S, Boufrioua G, Elalouani F, Barkane M, et al. Therapeutic hypothermia in asphyxiated neonates: Experience from neonatal intensive care unit of University Hospital of Marrakech. International journal of pediatrics. 2017;2017*. The measure of the estimate was RR. Your comment is correct and we have tried to amend the manuscript as per your comment. Thanks for your detail noticing of the manuscript.

**Reviewer comment:** 2. I suggest the authors also refer to the most recent guidelines from ILCOR (2020) regarding hypothermia treatment cooling to only be considered in neonatal care facilities with the capabilities for “multidisciplinary care and availability of adequate resources to offer intravenous therapy, respiratory support, pulseoximetry, antibiotics, anticonvulsants, and pathology testing”.

**Authors’ response:** thanks for supplying us with such a latest source which we have included the idea the manuscript (Back ground; page 4, Line 92-95) and in Discussion ; page 13, Line 280-283).

2. Some language issues

**Reviewer comment:** line 65: one of the strategies

**Authors’ response: amended; line 66**

**Reviewer comment:** line 113. What is "snowball search"?

**Authors’ response:** "snowball search" is to refer the following process. When we get an article with the outcome of interest of the meta-analysis we then look at all references of that article and if we get any **cross-referenced** article which include our outcome of interest we will download the PDF and again we look at its references this process continues until we didn’t get any new article. We tried to indicate this when we say snowball.
**Reviewer comment:** line 152: Instead of "the mistyping" perhaps better is "any mistyping"

**Authors’ response: amended; line 158**

**Reviewer comment:** Line 186: methods head cooling. Remove "head"

**Authors’ response: amended; line 192**

**Reviewer comment:** Line 198, comma lacking, base deficit

**Authors’ response: amended; line 204**

**Reviewer comment:** Line: 236 ischemia not eschemia

**Authors’ response: amended; 238**

**Reviewer comment:** line 238: methods

**Authors’ response: amended; 240**

**Reviewer comment:** 278: literature not literatures

**Authors’ response: amended; 283**

**Reviewer comment:** Conclusion: A well performed study, and now much improved. Please check the calculations mentioned above and change conclusions accordingly.

**Authors’ response:** we have amended the conclusion considering your comments in other parts of the manuscript. Line 308-320.

***Thanks in advance***
